# Cultural applicability and desirability of ‘Broodles’: The first serious game intervention for siblings of children with disabilities

**DOI:** 10.1016/j.pecinn.2024.100277

**Published:** 2024-03-26

**Authors:** Linda K.M. Veerman, Krister W. Fjermestad, Torun M. Vatne, Paula S. Sterkenburg, Suzanne D.M. Derks, Anjet A.J. Brouwer-van Dijken, Agnes M. Willemen

**Affiliations:** a Vrije Universiteit Amsterdam, Amsterdam Public Health, LEARN!, De Boelelaan 1105, 1081 HV, Amsterdam, the Netherlands; b University of Oslo, Boks 1072 Blindern, 0316, Oslo, Norway; c Frambu Resource Center for Rare Disorders, Sandbakkveien 18, 1404, Siggerud, Norway; d Bartiméus, Oude Arnhemse Bovenweg 3, 3941 XM, Doorn, the Netherlands; e Sibling Carers Community BRUS, Utrecht, the Netherlands

**Keywords:** Serious game, Siblings, Intellectual disability, User evaluation, Cultural applicability

## Abstract

**Objective:**

Serious games can serve as easily accessible interventions to support siblings of children with disabilities, who are at risk of developing mental health problems. The Dutch serious game ‘Broodles’ was developed for siblings aged 6–9 years. The current study aims to assess the cultural applicability, desirability, feasibility, and acceptability of ‘Broodles’ in Norway.

**Methods:**

Norwegian siblings (*N* = 16) aged 6–13 years and parents (*N* = 12) of children with intellectual disabilities assessed the game. Their feedback data from interviews and questionnaires were sorted using a model of engagement factors in serious games.

**Results:**

At pre-use, participants showed interest in the game, and after initial use the participants were overall positive about the format, content and objectives, including validation of emotions and recognition. The participants had suggestions for improved engagement and feasibility.

**Conclusion:**

The game was found to be culturally applicable, desirable and acceptable, although Norwegian translation is necessary for further evaluation. Recommendations to enhance engagement were provided, including suggestions to play the game with parents or in a group.

**Innovation:**

This initial assessment of the serious game Broodles in a non-Dutch setting shows promise for an innovative way of supporting siblings of children with disabilities.

## Highlights

• Few well-evaluated interventions for siblings of children with disabilities exist.
• The Dutch serious game ‘Broodles’ is a potential support intervention for siblings.
• User perspectives are essential in the cultural adaptation of serious games.
• Norwegian children and parents gave their initial impressions of ‘Broodles’.
• User feedback informs gaming-theory derived adaptation tips for the ‘Broodles’ game.

## 1 Introduction

Serious games are computer games with educative or therapeutic aims designed and presented in a playful manner. Serious games are increasingly being used in education and healthcare. They are engaging, can result in more effective learning than conventional instruction methods, and can overcome access barriers and care burdens [[1], [2], [3]]. Serious games can be used in raising awareness, detection, prevention, and treatment of health problems [4], and can take various forms, use different techniques and gaming elements [5]. Meta-analyses have shown that serious games are potentially effective in promoting skill development and mental health in children with and without disabilities [3,6]. A recent randomized controlled trial (RCT) found a serious game for children with autism spectrum disorder (ASD) to be effective for children's social-emotional skills [7]. However, few high-quality studies of serious games are available, little is known about long-term benefits, and more game research is needed, including engagement and feasibility research [3]. This paper presents initial user evaluations of a serious game for siblings of children with disabilities.

Siblings of children with disabilities represent an at-risk group [8]. Recent reviews and meta-analyses have shown that siblings have increased risk of mental health problems and decreased well-being compared to peers (*g* = 0.13–0.22) [[8], [9], [10]]. Siblings may also show more prosocial behaviour than controls [9]. Qualitative studies have reported that siblings experience daily challenges and conflicting thoughts and feelings, which affect their social relations [[11], [12], [13]]. Siblings often hide negative emotions from others [13], due to loyalty towards parents and the concept of double protection. “Double protection” means parents and children try to emotionally protect each other by avoiding talking about sensitive topics [14,15]. The impact of having a brother or sister with a disability on siblings' well-being is associated with a large number of interrelated risk and resilience factors on the individual, family, and structural level such as sibling executive functioning and coping skills, diagnosis type and symptom severity, socio-economic status, parental stress, and social support [10,[16], [17], [18], [19]]. Researchers have emphasised that siblings may benefit from preventive support to enhance their quality of life, well-being, and coping skills [11,20].

A recent meta-analysis [21] documented an increase in the number of interventions for siblings of children with disabilities since 2010. Mixed-methods evaluations have shown that these interventions can successfully target important sibling outcomes such as self-esteem, social support, and coping skills [22]. However, little profound evidence is available about the effectiveness and working mechanisms of these interventions [23,24]. Moreover, the vast majority of interventions for siblings are offered on-site by volunteers or care providers. Few evidence-based interventions are available to families [21], causing barriers regarding accessibility, affordability, and planning [25]. Online interventions with low involvement of providers can be beneficial to overcome these barriers.

This study concerned an online intervention, the serious game ‘Broodles’, created in co-creation with users by researchers from Vrije Universiteit Amsterdam in the Netherlands [26]. ‘Broodles’ targets 6 to 9 year old siblings of children with intellectual disability (ID) and/or visual impairment (VI). The aims of the game include: (1) recognizing, exploring and acknowledging complex thoughts and feelings about having a brother or sister with a disability, (2) strengthening active coping skills in dealing with these thoughts and feelings and with complex situations in the family, and (3) knowing that there are other siblings to prevent feeling they are alone. These aims are targeted through videos, reflective questions, mini-games about helpful thoughts and emotions, confirmations, and explanations. The components of the game tap into important risk and resilience factors associated with sibling well-being, including adaptive emotional functioning, coping skills, and social support [10,16]. The game incorporates important mechanisms in sibling interventions: ‘validation of feelings and experiences’, ‘increased communication with the parent’, and ‘respite’ [24]. The effectiveness of this intervention is currently studied in a Dutch RCT [26].

Few online sibling interventions and interventions for siblings below the age of 8 years exist [23]. Because a game can be used anywhere and is not dependent upon culturally specific health- and social services, it is easy to disseminate to other countries. However, little is known about cross-cultural differences in the well-being and experiences of siblings, let alone the impact of culture on the applicability of interventions [27]. Thus, there is a need for international cross-cultural validation and adaptation of this game. Cultural adaptation can be defined as *“the systematic modification of an evidence-based treatment (EBT) or intervention protocol to consider language, culture, and context in such a way that it is compatible with the client's cultural patterns, meanings, and values”* (p362) [28]. Studies have suggested that, when indicated, cultural adaptation could improve the effectiveness of mental health interventions within a cultural group [29]. This is a complex and lengthy process, that requires careful considerations in order to reach a good fit with cultural needs, without changing the core elements of the intervention. Therefore, one should first test the original intervention in a new context and identify which cultural adaptations are needed [30]. For digital health interventions, it is also important to assess the acceptability of the digital format in the new context, as this appears to be interrelated with engagement and effectiveness [31]. It is advisable to start with similar countries before expanding to more diverse cultures.

Therefore in the current study, we tested the Dutch-developed serious game ‘Broodles’ in Norway, to make a first inventory of facilitators and barriers that needs to be considered for cultural adaptation. We considered differences in the ‘macrosystem’ in these countries, *“the sociocultural environment, consisting of the cultural values, laws, customs, and resources of the context in which an individual develops”* (p309) [32], as this impacts sibling well-being. The Netherlands and Norway share similarities as European countries with a welfare state and well-performing health care systems [33]. Differences are present in the health and educational systems [34], attitudes, and resources, which can impact the feasibility of the learning goals of the game. For example, lower population density and larger rural areas decrease care accessibility in Norway [35]. More favourable in Norway are the parental work-care divisions [36] and participation rate of children with disabilities [37], and the fact that care services are legally required to provide support and information to siblings of children with illnesses [38]. Nevertheless, in both countries there is a lack of appropriate routine support for siblings, and support is mostly offered when difficulties arise and families request support [[39], [40], [41]]. Challenges in offering sibling support include shortages in personnel and financial resources, and access burdens.

This study's overall research question was: Can the serious game ‘Broodles’ be an easily accessible intervention for siblings in Norway? Cultural applicability, desirability and feasibility were assessed during group discussions and/or by playing parts of the game. Pre-use acceptability: the willingness to use the game, and initial use acceptance: the satisfaction about using the game, were also evaluated [42]. Acceptability and effectiveness of online interventions are related to engagement [31], i.e., the ‘extent of usage’ and the ‘subjective experience’ of using the intervention [43]. Therefore, factors and game components that contribute to engagement, and thus obtaining learning goals, were investigated, using Vacca et al.'s model [5] formulated for serious games, based on the ‘Elemental Tetrad of Games' [44].

This paper has relevance for potential implementation in Norway, but also provides unique insights to the sibling user perspective that is relevant beyond these two cultures and this particular game.

## 2 Methods

### 2.1 Serious game ‘Broodles’

**Fig. 1:**
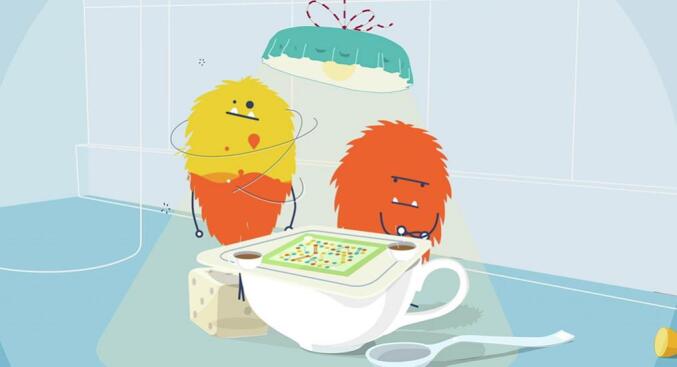
Broodle Creatures.

‘Broodles’ is a single player, narrative, web-based game which takes its name from fantasy creatures called “Broodles” (see Fig. 1). The game was created according to gaming theory, incorporating self-determination theory [45] and a co-creation model [46]. The gaming elements, including feedback and rewards, contribute to users' motivation and engagement, supporting effective learning [47]. The content and learning objectives are based on previous findings about sibling quality of life [20], important sibling intervention elements [24], and a sibling support book [48].

The gaming elements and structure of the game are based on the serious game ‘You & I' [46], and adapted to siblings. This was done in close collaboration with young siblings, parents, an advisory board of experts in the field, and adult siblings, including a sibling co-researcher (ABvD). Although the target group is siblings aged 6 to 9 years old, we also included slightly older children (up to 11 years) in the creation and testing phase, to receive more detailed input and feedback. Different groups of siblings were involved throughout the creation process. At the start of the project, nine children (of which three had a sibling with ID) provided input about their gaming interests and experiences with their sibling. Next, upon the creation of the game, the ‘young sibling user panel’ was formed, including five children (2 boys, 3 girls) aged 6 to 11 years with a brother or sister with ID, ranging from mild to profound ID, and a range of comorbid conditions, including VI, ASD, physical disability, or Down Syndrome. They were recruited through social media and the researchers' professional network. Over a period of six months, the children provided input and feedback in two group- or individual sessions (one online, one on site), and four rounds of written feedback and input on different elements of the game (e.g. storylines, visuals). They, for example, chose the rewards, initiated to change the feedback sounds, and posed the idea to include a song. The children were asked to provide general feedback and specifically comment on attractiveness, recognizability, and comprehensibility. They also shared their sibling-experiences in videos in the game. When the full game was created, four children of the sibling panel and three additional children tested the game and provided written and verbal feedback. The advisory board gave input and feedback on the game's content and format in multiple meetings.

**Fig. 2:**
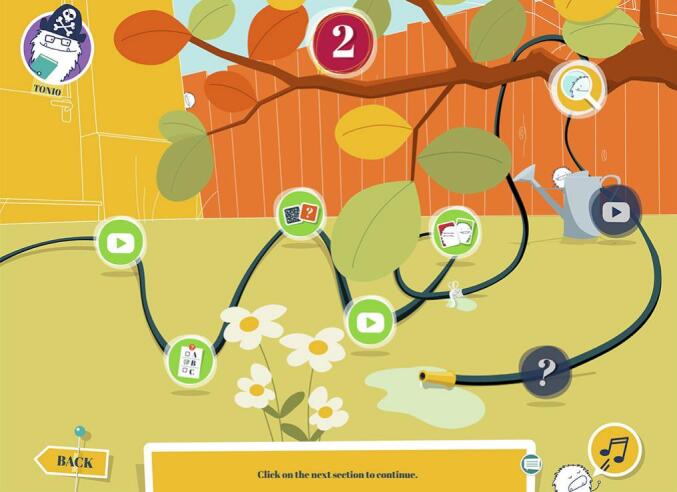
Level Overview.

**Fig. 3:**
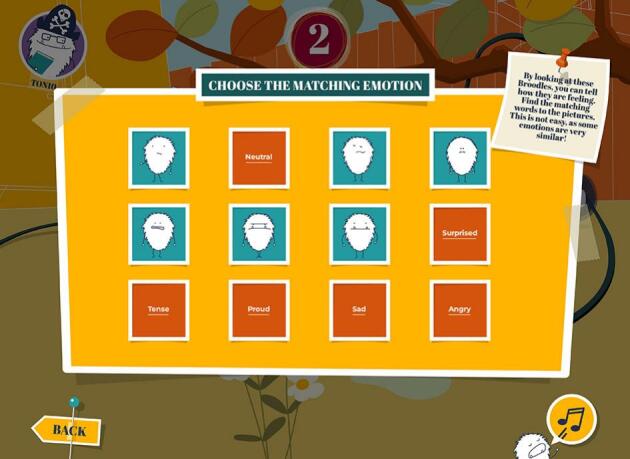
Emotions Memory.

The game comprises eight levels that each take about 20 min to complete (see Fig. 2). One of the levels is designed to be played with a friend, the other levels can be played alone. Each of the levels has a different theme, stemming from the ‘domains of sibling quality of life’ [20], and comprises the same eight game elements, including: two animation videos to enter the ‘Broodles’ world, two quizzes with questions about the videos and personal experiences, a lived-experience video, and three mini-games: emotions memory (see Fig. 3), a helpful thoughts game, and a hidden object game.

The set up, order, and content are all meant to contribute to recognition and learning coping skills. Furthermore, the feedback that is provided in the game focusses on affirming and normalizing thoughts and feelings, thus encouraging the player to talk about these and sooner ask for help or support. Interactive and playful elements are included, such as Broodle avatar choices, a Broodle song, and rewards in a virtual scrapbook. The game is not individually adaptable; all children are given the same materials. With the aim to connect the game even more to daily life and experiences, offline worksheets are provided. These consist of a short summary of the storyline, a helpful thought, a tip or trivia, and an exercise linked to the theme of the level. The worksheets are to be completed after each level, optionally with a parent.

The game can be played without assistance. Because siblings are encouraged by game contents to ask for help, parents receive a short guide with the background, aims and contents of the game, tips to support the sibling and references to other sibling support resources. This guide was based on research and resources [20,48], and written by the researchers, the adult sibling co-researcher, and sibling experts from the advisory board. Researchers can request more information about the game, worksheets and parental guide from the first author.

‘Broodles’ is the first serious game for siblings of children with disabilities. The game was translated from Dutch into English by a bilingual Dutch/English-speaker from the production team, who also wrote the original storylines. The English translation was made prior to the conceptualization of this Norwegian evaluation, but this was the first time it was used. A Norwegian translation has not been made yet. Researchers and parents translated the content to Norwegian while children were playing.

### 2.2 Sample and procedures

This study was approved by the Department of Psychology's internal research ethics committee at the University of Oslo (#28031280). The study was also registered at SIKT, a Norwegian privacy institution that assessed the study protocol, to ensure that personal data is processed according to privacy legislations (# 805047).

Before the start of the study, a Norwegian clinical researcher reviewed all the materials to examine if it would be a suitable intervention to test with Norwegian families and identify possible barriers or culturally sensitive elements (e.g., as described in the ecological validity model [49]). Two Norwegian researchers involved in this study (KW, TV) reviewed part of the materials as well.

Participants were recruited both through websites or newsletters of Norwegian user organizations for families with children with ID and/or VI, and the family courses at *Frambu resource centre for rare disorders*. Siblings and their parents were eligible for participation when they were 6 to 10 years old, had a brother or sister with ID and/or VI, and they did not have a severe disability or illness themselves. One 13-year-old boy and his father participated in a group meeting at Frambu as they were part of the family course. We included their evaluations, because the father reflected on the intervention thinking of when his son was younger, and the boy contributed to confirming the appropriate target age range.

Informed consent was obtained from one or both of the parents, giving consent for their own and their child's participation in the study. The participant information included verbal information provided by a researcher to the parent and written information in separate letters for parent and child. The study was conducted in September and October 2023.

**Fig. 4:**
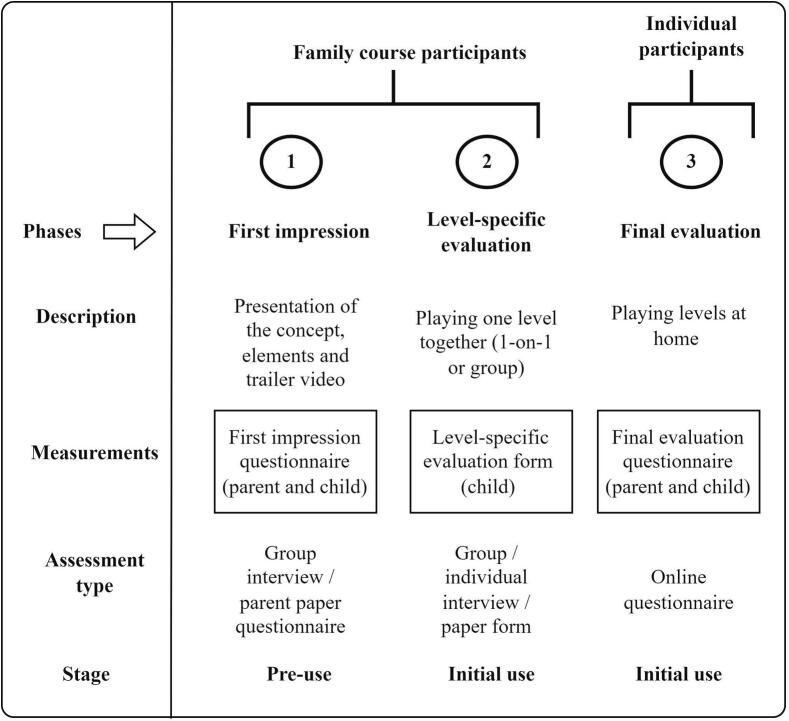
Overview of Evaluation Phases.

Participants could take part in three different test phases (see Fig. 4) in which they evaluated different parts of the serious game, additional worksheets and a parent guide: (1) first impression phase, (2) level-specific evaluation phase, and (3) final evaluation phase. The first two phases, due to practical logistical reasons, were only available for the families that followed a course at Frambu. This was done in three different groups. Due to staff availability, differences in group size, and English proficiency of the participants, some differences in the procedures occurred, including the use of paper questionnaires versus interviews, and playing in a group versus playing one-on-one with a researcher.

All of the interviews were audiotaped using a voice recording application. The participants that played the game at home completed online questionnaires through Qualtrics [50].

### 2.3 Measurements

Three evaluation questionnaires were developed by the research team, for the different phases of the evaluation procedure. The First Impression Questionnaire was tested with two Norwegian children (9 and 11 years) and the Level-Specific Evaluation Form and Final Evaluation Questionnaire have been tested with the Dutch young sibling user panel. No feedback was provided by these children that lead to changes in the questionnaires. Questions regarding cultural applicability were added to the current questionnaire. The used questionnaires can be found in Appendix A.1–A.4.

#### 2.3.1 Phase 1: First impression questionnaire

To evaluate the concept of the game, illustrated by a PowerPoint and trailer video, parent and child report questionnaires were used. The child questionnaire comprised eight open-ended questions and the parent version consisted of seven open-ended questions regarding first impressions of the visuals and pre-use acceptability of the concept and purpose of the game. An example question is: ‘What is your first impression of what the game looks like?’

#### 2.3.2 Phase 2: Level-specific evaluation form

To evaluate the first level of the game, a short evaluation form was used, comprising four statements that the children were asked to complete: (1) ‘I liked this…,’ (2) ‘I did not like this or this was strange to me…, ’ (3) ‘I did not understand this…,’ (4) ‘I think the worksheet and the task I had to do was…’ In addition, the form included two open-ended questions: ‘What did you learn from the game and worksheet?’ and ‘What did you miss in the game or worksheet?’ The children could write down their impressions on the form. In the case of interviews, follow-up questions were used to specify the child's answers.

#### 2.3.3 Phase 3: Final evaluation questionnaire

To evaluate the full intervention, an adapted version of the post-test evaluation questionnaire used in Veerman et al. [26] was completed by the participating children and parents. This questionnaire was based on the Social Validity Scale (SVS) [51]. The child report version included eight closed evaluation questions with a 5-point Likert scale with smileys and 18 open-ended questions. The parent report version included 26 open-ended questions. The questions were focused on evaluating the desirability, feasibility, attractiveness, and usefulness of the different elements of the intervention. Parents and children (with assistance of their parent) could type their impressions in text entry boxes. An example question is: ‘Would you recommend this intervention to other families? Why (not)?’ The internal consistency of the closed items of the SVS questionnaire was acceptable (α = 0.76). This should be interpreted with great caution, as only four participants filled out this questionnaire.

### 2.4 Data analysis

Two researchers (KF, LV) summarized the user evaluations, using the model of engagement factors in serious games, as proposed by Vacca et al. [5]. This includes: (1) ‘Game elements’, the different characterizing parts and mechanisms in the game, (2) ‘Technology’, the way the game works and its usability, to which we added ‘Format’, to also include broader comments about the way the game is arranged, (3) ‘Narrative’, the storyline and situations represented in the game, (4) ‘Aesthetic’, the look and feel of the game, and (5) ‘Purpose’, the perceived learning outcomes of the game.

This procedure included listening to the voice recordings and writing down the main comments by both researchers together; extracting comments from the written forms and online questionnaires; individually categorizing the comments from the three phases to the model factors, and at the same time labelling them as positive, negative/improvements, or neutral/ambivalent; grouping comments; individual re-evaluation and refinement of the categorization and grouping of comments. In between these steps the two researchers evaluated the participants' feedback with a third researcher (TV) that was involved in data collection. All co-authors, including a sibling co-researcher (ABvD), revised the categorization and grouping.

In addition, general comments about cultural applicability, desirability and feasibility were summarized separately. Finally, item and total means on the closed SVS-questions were reported and used to check the interpretation of the overall impression based on the qualitative evaluations.

## 3 Results

In total, 14 families, including 16 children and 12 of their parents participated in the study (see Table 1). This included children age 6 to 13 years (*M* = 7.88, *SD* = 1.89, 63% boys). Parents (58% mothers) were between 33 and 51 years old (*M* = 41.36, *SD* = 5.64). Fifty-five percent of the parents had more than four years of higher education. The siblings of these children were between the age of 2 and 14 years (*M* = 7.47, *SD* = 3.29, 1 missing), and had ID due to various genetic disorders, such as Prader-Willis syndrome, Tuberous Sclerosis Complex, and Down Syndrome. The user evaluations of these families are summarized in Table 2, sorted by engagement factors [5].

**Table 1:** Overview of Participating Families.

Family	Group	Parent	Child (age)	Evaluation phase	# levels
1.	1	Father	Boy (13)	1. First impression	0
2.	1	none*	Boy (7) Girl (10)	1. First impression 1. First impression; 2. Level-specific evaluation	0 1
3.	1	Mother	Boy (7)	1. First impression; 2. Level-specific evaluation	1
4.	2	Father	Boy (6)	1. First impression; 2. Level-specific evaluation	0.5
5.	2	Mother	Girl (7)	1. First impression; 2. Level-specific evaluation	0.5
6.	2	Mother	Girl (7)	1. First impression; 2. Level-specific evaluation	0.5
7.	2	none*	Girl (9)	1. First impression; 2. Level-specific evaluation	0.5
8.	2	Father	Girl (7)	1. First impression; 2. Level-specific evaluation; 3. Final evaluation	2
9.	3	Mother	Boy (8)	1. First impression; 2. Level-specific evaluation	1
10.	3	Mother	Girl (9)	1. First impression; 2. Level-specific evaluation	1
11.	3	Father	Boy (7)	1. First impression; 2. Level-specific evaluation	1
12.	*N/A*	Mother	Boy (6) Boy (7)	3. Final evaluation 3. Final evaluation	1 1
13.	*N/A*	Father	Boy (6)	3. Final evaluation	6
14.	*N/A*	Mother	Boy (10)	3. Final evaluation	1

*Note.* Siblings that played one level played only the first level. The children in the second group played only half of the first level, due to restricted time. Both boys in Family 12 completed one evaluation questionnaire together. *Parent did not participate.

**Table 2:** *User Evaluations per Factor Related to Engagement in Serious Games* [5].

	Phase 1: Pre-use	Phase 2 & 3: Initial use
Game elements	Child comments: + Quizzes + Mini-games, such as memory + Choose your own avatar⁎ - Worksheets; e.g. ‘boring’ - Questions and finding Broodles looks bit difficult⁎ - Needs more choice and challenging elements⁎ - Sibling-related (personal) videos and questions are ‘boring’⁎ Parent comments: + Interactive and challenging elements + Worksheets + Good selection of game elements⁎ - There should not be “right” and “wrong” answers to quizzes⁎ - Difficult to find the balance between making it attractive (fun) while relevant (sufficiently challenging)⁎	Child comments: + General comments: “Everything”, “Many different things” + Hidden-object game: ‘catching/hunting Broodles’ + Quizzes, including True/False questions + Videos of other children and Broodles animation videos + Helpful and fun to have worksheets + Friendship topics: ‘I liked that they became friends⁎ - Hard to distinguish emotions in memory game - More motivational cues e.g. points or battles with others - More interactive elements: less/shorter videos, more mini-games - Too verbal: too many questions, too much talk - Questions can be hard for younger children - Worksheet was boring; did not like the task - More quiz answers should be “correct”⁎ - Mini-games should be earlier in the level⁎ +/− Right or wrong answers to quizzes make you think, but can cause frustration⁎ Parent comments: + Good variety of game elements + Hidden-object game was most fun + Most useful were the videos of other siblings + Elements regarding coping with thoughts, feelings and difficult situations + Broodles animations “catchy”⁎ - Hard to distinguish emotions in memory game - More “find Broodles”-type tasks to maintain attention - Worksheet was too difficult for young age⁎
Technology/Format	Child comments: + Short duration of levels positive as this will prevent boredom - Too short, too few levels +/− Ambivalence about playing alone versus with parents/others Parent comments: + Duration and amount of levels are appropriate + Game format is appealing and fits the target group + Playing alone is fine - Playing together with the parent would be better - Desirable to have the option to see the child's quiz answers⁎	Child comments: + The sound effects - Technical issues with videos and hidden-object game (WiFi or device issues) - Would prefer to play with others in group, mother/father, or sibling with diagnosis (over playing alone) - No need for “next” button, should be automatic⁎ +/− Ambivalence about sound effects with “wrong” answer Parent comments: + In Norwegian, playing alone would be fine - Parent involvement desired, e.g. to relate it to their own live or to explain about difficult or nuanced topics - A mobile app might work better, it is harder to focus on right tasks on a tablet for younger children⁎ - Something to keep it relevant in the real world is missing⁎
Narrative	Child comments: + Children can relate to other siblings in the videos; this adds substance; “real life examples” are good; makes it recognizable + Videos with the real siblings were less childish; seemed more real⁎ + Recognition elements (associations to own situations)⁎ - Should be about the same diagnosis as my brother/sister⁎ - Confusion about fantasy elements⁎ Parent comments: - May seem boring to some children⁎ +/− May be harder to relate to other disorders than own but also good to see diversity⁎	Child comments: + Recognition of situations in the game⁎ + You can learn from the children in the videos⁎ + Message is to help and be aware of feelings⁎ - More exciting elements could be added, e.g. space theme⁎ - Confusion and frustration about the functioning level of sister in the game being better than of my brother/sister with the same diagnosis⁎ - Start video introducing Broodles takes long to get to the point⁎ - Missing the ability to add own perspective instead of answering based on Broodles' experiences⁎ +/− Struggled to recognize situations or relate it to own life⁎ Parent comments: + Good to see children in the videos with the same level of functioning⁎ - Should include the perspective of the child with ID (for the sibling to learn)⁎ - More variance in functioning level of portrayed children in the game, to increase recognizability - Can be hard to keep attention, e.g. too slow, too much talk
Aesthetic	Child comments: + Nice videos + Broodle figure details, such as ‘embroodlement’ + Looks “cool”, “funny”, “pretty”, “nice” + Nice colors⁎ - Looks boring or childish, needs more excitement - Hard to tell Broodles figures from one another, they all look similar⁎ - Figures may be scary to younger kids⁎ - Some boards are a bit messy and confusing⁎ - Suggested changes including space elements, more symbols use (hearts, stars etc) and more varied animations (e.g. more animals)⁎ +/− Questions concerning age-appropriateness of aesthetics, different opinions about who it fits best for⁎ Parent comments: + Positive, appealing graphics + Looks educational⁎ - Color and figure scheme may be less exciting/appealing than the games they usually play⁎ +/− Puzzled at how similar Broodles look apart from different colors⁎	Child comments: + Broodle figures are nice and fun + Nice colors - The Broodles should be more different from each other, so you can see who is who. +/− Some puzzlement at how the Broodles look, e.g. why they have sharp teeth⁎ +/− Ambivalence regarding how “real” or how “fantasy”-based the game is⁎ Parent comments: *None*
Purpose	Child comments: + Learn about the disease – sibling-specific/general + Learn how to cope with or help your brother/sister + Recognition in other siblings (in game) + “We play it to learn, not for fun”⁎ - Do not think they can learn from it⁎ Parent comments: + Learn that they are not alone/community belonging + Gives chance to open up/talk/reflect⁎ + Learn about own feelings/understand self⁎ + Normalize feelings⁎ +/− Should also include positives/ability to see good traits in brother/sister with disorder⁎	Child comments: + You can learn how to cope/deal with/tolerate/help your siblings and how to behave around them and other children + Learning I am not alone/there are others with a brother/sister with a disability + Learn about feelings⁎ + “I learned that it is OK to have a brother with a diagnosis”⁎ + Learn to not give up, try more/harder⁎ - Think they did not learn much in general, or specifically about feelings or dealing with difficult situations +/− Ambivalence about if and what learnt Parent comments: + Gain better understanding of own brother/sister + Gives chance to open up/talk/reflect + Learn that he/she is not alone + Helps to process thoughts and feelings and learn you are allowed to feel them⁎ + Can enhance positive processes that are already present in the family⁎ + Learn new ways of thinking/coping with situations - Balance learning and the child's sense of “over”-responsibility⁎ - Did not learn that much, because it was too difficult for his age⁎ +/− Purpose of the animation videos is unclear⁎

*Note.* Comments categorized as positive are indicated with ‘+’, comments categorized as negative or suggestions for improvement are indicated with ‘-’, and comments categorized as neutral or ambivalent are indicated with ‘+/−’.

⁎ Comments mentioned by one participant or in one group interview.

### 3.1 Cultural applicability, desirability and feasibility

The Norwegian sibling researchers who reviewed the intervention, indicated that portrayed persons, images, content en context seemed appropriate to the Norwegian culture. Regarding language, apart from translation to Norwegian, it was noted that some terms were unfamiliar (e.g., ‘sibling carer’). Some pedagogical comments were made, for example that some phrasings might appear overly ‘direct’ to Norwegian children. The participating children and parents did not mention any elements that seemed culturally inappropriate and they were positive about translating the game to Norwegian. They suggested to adapt the names of the game, characters and children to fit the Norwegian language.

Considering desirability, most children and parents mentioned that, based on their first impression, they wanted to play the game. After playing part of the game, they indicated that they wanted to continue playing and would recommend the game to others. The one 13-year-old boy was not interested in playing, as it was not appropriate for his age. Participants mentioned that the game is appealing and important to children in the target group, but that some might find it less appealing than ‘ordinary’ games. One parent underlined that the desirable timing to use the game can differ per family, for example it might not be desirable to use when the child with a disability does not yet themselves know about their condition.

Finally, regarding feasibility most participants were positive, but some parents mentioned that not all children might be able to play it alone and need more help, including: motivating to play the game and maintain attention, supporting with and explaining difficult topics in the levels, and following up conversation about the topics. However, some parents indicated that they might lack time and energy to provide their children with this support. As for the parent guide, most parents mentioned that it is useful and the appropriate length, but some think it is quite extensive and contained a significant amount of information to manage or address.

### 3.2 Acceptability and engagement factors

The participants were generally positive about the gaming elements, format, aesthetic, narrative and purpose of the game, both based on their first impression (pre-use acceptability) and after playing a part of the game (initial use acceptance). The interactive mini-games were most appreciated for its engaging purpose, as well as elements in the game that were recognizable, such as the videos of other siblings. Suggestions for improvement included shortening some elements, expanding with more challenges or additional mini-games. Many children expected that they would learn about diseases and disabilities, but after playing part of the game, the participants mentioned that they learned skills as intended by the objectives of the game (See Table 2).

The qualitative findings are in line with the scores of the children (*n* = 4) that completed the closed SVS-questions. The mean score across all items was 3.22, with item means ranging from 2.00 to 4.25, where 3 represents the neutral score. Siblings were most positive about the minigames (*M* = 4.25, *SD* = 0.50), and all siblings would recommend the game to other siblings (*M* = 4.00, *SD* = 0.82). They were most critical about the length and number of levels (*M* = 2.00, *SD* = 0.82), the idea of playing it without the parent, although this was because of the English language (*M* = 2.00; *SD* = 1.16), and the worksheets (*M* = 2.25; *SD* = 1.50). Finally, siblings were neutral (3) to very positive (5) about the game in general, the overall aesthetic, and the videos (*M* = 3.75, *SD* = 0.96).

## 4 Discussion and conclusion

### 4.1 Discussion

The aim of the current study was to acquire user perspectives, to provide exploratory evidence that the serious game is culturally applicable, desirable, feasible, and acceptable in Norway. Suggestions for improvement have been identified that need to be considered when translating and further testing this serious game in Norway.

#### 4.1.1 Cultural applicability, desirability, feasibility and acceptability

First, the serious game ‘Broodles’ appears culturally applicable in Norway, based on a first cultural screening of the intervention by a Norwegian researcher and preliminary testing with users. Other than translating the content, pedagogical phrasing, and names to Norwegian, no larger cultural adaptations seem necessary. Next, desirability was supported, as parents and siblings found the serious game a good initiative, that they would make use of and would recommend to others. This study showed the appeal of the format of a serious game to children, as was found in previous research [3]. Pre-use acceptability and initial use acceptance were confirmed, as parents and siblings indicated that the game's format and aesthetics were appealing, the game elements and narrative were engaging, and its purpose was clear. However, users' expectations should be managed by providing information about the game's goal, as in this study most siblings had different pre-use expectations. They expected to learn about the disability, which makes sense as, opposed to the current intervention, most sibling programs also focus on knowledge about the condition (e.g., Zucker et al. [52]). After playing a part of the game, they indicated that the game contributed to the intended learning objectives regarding emotional and coping skills, knowing you are not alone, experiencing validation of emotions, and increased communication between the sibling and the parent, which have been found to be important mechanisms in sibling intervention [24]. The game could help siblings, who often tend to hide their feelings [13], to open up and start talking about it with their parent.

Previous sibling intervention studies suggested that families might not participate in support programs due to practical issues (e.g., costs, lacking time, conflicts in planning) [24]. Parents of children with disabilities are highly burdened and can be exhausted from caregiving tasks [53]. We expected that the current intervention has higher feasibility than existing programs, because it has greater flexibility in where, how and when it is used. Still, parents mentioned that it was sometimes hard to find the time to play the game, although this is likely because parents needed to help translating the game. However, even when siblings can play the game alone, all siblings will demand some time from their parents when playing, as it aims to help siblings to talk more about their inner world and to ask for help sooner. Moreover, parental involvement is an important element of sibling support programs [25,54].

#### 4.1.2 Suggestions for improvements

Suggestions for improvements were made, which mostly reflected children's wishes to increase enjoyment and excitement of the game. Although children prefer games to be fun, it is not the main predictor of success of an intervention [55]. Finding the right balance between motivation, engagement, and educational elements is a challenge that is common in serious game design [56]. A Norwegian study showed that gaming preferences of children are diverse, and related to several factors such as age, where some prefer more active games and others prefer learning games, indicating that a game can never be attractive to everyone [57]. Also, for some children, dismissing elements as ‘boring’ may reflect a level of emotional avoidance of recognized own difficulties [58], or it could be part of the loyalty or double protection scheme within the family.

As for the narrative, to a few children some storylines were less recognizable, because it did not correspond to the functioning level of their sibling. Although recognition of situations in the game is important to feel heard and therefore supported [24], noticing differences between the siblings in the game and their own experiences, could also lead to meaningful conversations with the parent about this topic. When sibling play the full game, they may recognize more situations, as the game discusses different themes that are relevant for siblings across different disorders [20].

#### 4.1.3 Limitations

The current study has limitations. First, although it is of importance to test a translated version of the intervention to adequately explore its cultural applicability, the current study used an English version of the game [30]. As a result, a parent or researcher needed to translate (parts of) the game to Norwegian, causing parts of the narrative to get lost in translation. Some children mentioned this made the game less appealing, which may have influenced their evaluations.

Second, most families participated in this study during a support week, including the SIBS parent-sibling group intervention [59]. Therefore, they were likely more aware of siblings' support needs, and possibly more positive about the desirability of the game than families that have not received such support yet.

Third, the methodology of the current study only provides exploratory findings and does not provide quantitative or in-depth qualitative evaluations of the game. Most children only evaluated a small part of the game, and most parents only provided their first impression of its concept. Therefore, some families might have been more positive, because they have inflated expectations of the game, whereas other families might have been more negative, because they have not played the levels with themes that are more recognizable to them. Generalizability of the results is restricted, as we used a small sample size and did not further investigate important demographics, such as socio-economic status, disability type, comorbidities, and symptom severity, and the siblings' own (subclinical) neurodivergence [10,16]. Therefore, this study does not provide insight into which families might be more positive about this intervention, or might benefit more. Moreover, the current study does not provide enough evidence to claim cross-cultural validity of the intervention. A next step is to test a Norwegian translation in a larger sample, and investigate differences between families with different demographic characteristics.

Finally, the intervention itself has limitations. Although it does include a parent guide, some parents might need more support. For example, in communicating with their child and regarding family processes, such as learning about and having to come to terms with the diagnoses, and experiencing grief. Other sibling interventions with a parent component, for example as designed by Vatne et al. [59], might therefore be more beneficial to some families. In addition, the game does not provide psycho-education about the disability, which is important in promoting sibling well-being [60]. However, support needs differ between families and over time, and thus tailored support, and possibly combining interventions, is advisable [22].

#### 4.1.4 Future directions

This study demonstrated that ‘Broodles’ can be used in multiple ways to fit siblings' individual preferences and needs, which should be further investigated. Playing the game alone is fine for some, but not desirable for (younger) children that need more assistance. Playing together might be beneficial, as it makes parents more aware of their child's experiences and creates an opportunity for communication and support. The game could also be played together in a group of siblings with a teacher or therapist. This could increase effectivity compared to playing alone, because children can engage in other learning activities as well, such as reflecting on the topics together [1]. However, the development of the game was done with the knowledge that in the Netherlands – and equally in Norway – support groups are hardly available for the youngest siblings [40,41]. It would be important to further research how the game may be a driver for the rise of sibling interventions in the age-group of 6 to 9 years, or even if new siblings are reached that would normally not enroll or cannot take part in sibling groups due to access burdens. Moreover, it needs to be examined how ‘Broodles’ could fit in with other existing resources for families of children with disabilities, possibly as part of a stepped-care approach.

Future studies could also focus on assessing the cultural applicability of the game in non-European countries and non-western cultures, to investigate its global potential. Similarly, it is worth investigating the suitability for siblings of children with other chronic conditions, as it addresses topics and focuses on learning objectives that are relevant to siblings regardless of diagnosis [22].

### 4.2 Innovation

‘Broodles’ is the first serious game for siblings of children with disabilities in the world. This novel healthcare approach has shown promising results in promoting social-emotional skills and mental health of children [3,5,6]. As serious games are appealing, accessible and low cost, it overcomes the barriers that have been identified in existing sibling support programs and fits the existing need for preventive programs available for widespread use. The strong user perspectives' approach in the development of the game is a key innovative element as well.

### 4.3 Conclusion

The current study provides preliminary evidence that the serious game ‘Broodles’ is culturally applicable, desirable and acceptable to Norwegian families with a child with ID. User evaluations of young siblings and their parents indicate that the game is perceived as appealing, and that learning objectives of the game are targeted. The study provided new insights in potential settings to offer the game, for example as part of a sibling group, or playing together with a parent.

The results of this study indicate that it would be useful to take further steps in translating ‘Broodles’ to Norwegian, and examine different ways of using the game as a support resource, as well as its effect on siblings' wellbeing.

The following are the supplementary data related to this article.

Supplementary Appendix A.1 First Impression Questionnaires 'Broodles'. Supplementary Appendix A.1

Supplementary Appendix A.2 Level-Specific Evaluation Form 'Broodles'. Supplementary Appendix A.2

Supplementary Appendix A.3 Final Evaluation Questionnaire 'Broodles' - Child Version. Supplementary Appendix A.3

Supplementary Appendix A.4 Final Evaluation Questionnaire 'Broodles' - Parent Version. Supplementary Appendix A.4

## Funding

This work was funded as part of the Academic Lab “Social Relations and Attachment” (project number 641001101) by The Netherlands Organization for Health Research and Development ZonMw, Den Haag, the Netherlands. In addition, the first author (LV) received additional funding for her stay in Norway, including a PhD Travel Fund from the Faculty of Behavioral and Movement Sciences of Vrije Universiteit Amsterdam, and an Erasmus+ Traineeship Grant. The funding sources did not have any involvement in this study.

## CRediT authorship contribution statement

**Linda K.M. Veerman:** Writing – original draft, Visualization, Project administration, Methodology, Investigation, Funding acquisition, Formal analysis, Data curation, Conceptualization. **Krister W. Fjermestad:** Writing – review & editing, Writing – original draft, Visualization, Supervision, Resources, Project administration, Methodology, Investigation, Formal analysis, Data curation, Conceptualization. **Torun M. Vatne:** Writing – review & editing, Validation, Resources, Project administration, Methodology, Investigation, Conceptualization. **Paula S. Sterkenburg:** Writing – review & editing, Validation, Supervision, Methodology, Funding acquisition, Conceptualization. **Suzanne D.M. Derks:** Writing – review & editing, Validation, Methodology, Conceptualization. **Anjet A.J. Brouwer-van Dijken:** Writing – review & editing, Validation, Conceptualization. **Agnes M. Willemen:** Writing – review & editing, Validation, Supervision, Methodology, Conceptualization.

## Declaration of competing interest

The authors declare the following financial interests/personal relationships which may be considered as potential competing interests: Linda K. M. Veerman reports travel was provided by European Commission. Anjet A. J. Brouwer-van Dijken has minimal competing interests, because she may gain financially in the sales of her book for siblings that is referenced in this article and in the intervention, and was used as the basis of the game. However, she did approve to use the content of the book free of costs in the game, in the worksheets and for the parent guide. If there are other authors, they declare that they have no known competing financial interests or personal relationships that could have appeared to influence the work reported in this paper.
