# Supplementary material for: Cultural applicability and desirability of ‘Broodles’: The first serious game intervention for siblings of children with disabilities
Source: PEC Innov. 2024 Mar 26;4:100277. doi: 10.1016/j.pecinn.2024.100277 (PMC11000116; doi:10.1016/j.pecinn.2024.100277)
Supplement: Supplementary Appendix A.1 — First Impression Questionnaires 'Broodles'. [file mmc1.pdf]

Serious Game ‘Broodles’

First Impression Questionnaire – Child Version

1. What is your first impression of what the game looks like?
    - How the game looks in general;
    - The graphics of the creatures called Broodles;
    - The video’s of the children and what they tell;
    - The quizzes;
    - The different mini-games: emotion memory, helpful and non-helpful thoughts, hidden object game.

2. Is there anything in the game that seems strange to you?

3. Was it possible for you to understand what was said in the game?

4. Would you want to play this game? Why (not)?

5. Do you think you could learn something from this game?
    - In understanding your brother or sister;
    - About your thoughts and feelings;
    - About how to deal with difficult situations with your brother or sister.

6. What do you think about playing the game alone, without your parents?

7. What do you think about making worksheets after each level, with your parents?

8. What do you think about the duration (20 minutes) and amount of levels (8 levels)?

Serious Game ‘Broodles’

First Impression Questionnaire – Parent Version

1. What is your first impression of what the game looks like? Do you think your child would like this?
    - How the game looks in general;
    - The graphics of the creatures called Broodles;
    - The video’s of the children and what they tell;
    - The quizzes;
    - The different mini-games: emotion memory, helpful and non-helpful thoughts, hidden object game.

2. Is there anything in the game that seems strange to you?

3. Would you want your child to play this game? Why (not)?

4. Do you think your child could learn something from this game?
    - In understanding their brother or sister;
    - About their thoughts and feelings;
    - About how to deal with difficult situations with their brother or sister.

5. What do you think about playing the game alone, without parents?

6. What do you think about making worksheets after each level, with you/the other parent?

7. What do you think about the duration (20 minutes) and amount of levels (8 levels)?
