# Supplementary material for: Cultural applicability and desirability of ‘Broodles’: The first serious game intervention for siblings of children with disabilities
Source: PEC Innov. 2024 Mar 26;4:100277. doi: 10.1016/j.pecinn.2024.100277 (PMC11000116; doi:10.1016/j.pecinn.2024.100277)
Supplement: Supplementary Appendix A.2 — Level-Specific Evaluation Form 'Broodles'. [file mmc2.pdf]

1

Start + Level 1 Broodles

I liked this

I did not like this or this was strange to me

I did not understand this

I think the worksheet and the task I had to do was

What did you learn from the game and worksheet?

What did you miss in the game or worksheet?

Serious Game ‘Broodles’

Level Specific Evaluation (follow-up questions)

- What did you (not) like about:
  - The video’s of the Broodles
  - The video’s of the siblings
  - The quizzes
  - How the game looked
  - The mini-games, including emotion memory, helpful and non-helpful thoughts, hidden object game
  - How long it took to complete the level

- What did you think was strange about:
  - How it looked
  - What was said

- What did you learn from this level?
  - About your brother/sister
  - About your thoughts and feelings
  - About how to deal with situations

- What did you miss in this level?
  - What game element should have been there?
  - Which information would you have wanted about this topic?
