# Supplementary material for: Cultural applicability and desirability of ‘Broodles’: The first serious game intervention for siblings of children with disabilities
Source: PEC Innov. 2024 Mar 26;4:100277. doi: 10.1016/j.pecinn.2024.100277 (PMC11000116; doi:10.1016/j.pecinn.2024.100277)
Supplement: Supplementary Appendix A.3 — Final Evaluation Questionnaire 'Broodles' - Child Version. [file mmc3.pdf]

Serious Game 'Broodles'

Final Evaluation Questionnaire – Child Version

Hi! Great that you are joining this study and played the game Broodles! I will ask you a few questions about the game. The questions are about what you think about the game. There are no right or wrong answers. You can tell everything you want.

1. How much did you like playing the game Broodles?

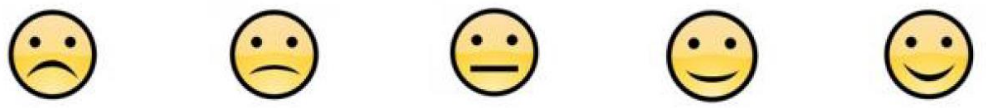

2. What did you like about playing the game?

3. What did you not like about playing the game? How could this be better?

4. How much do you like the way the game Broodles looks?

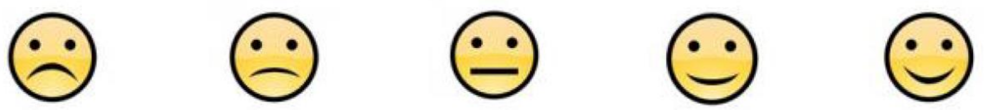

5. What do you like about the way the game looks?

6. What do you not like about the way the game looks?

7. How much do you like the videos in the game Broodles?

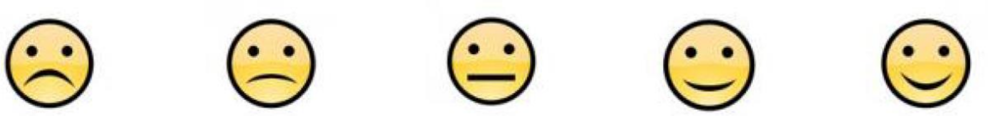

8. What do you like about the videos?

9. What do you not like about the videos?

10. How much do you like the minigames (quizzes, emotion memory, helpful thoughts game, hidden object game) in the game Broodles?

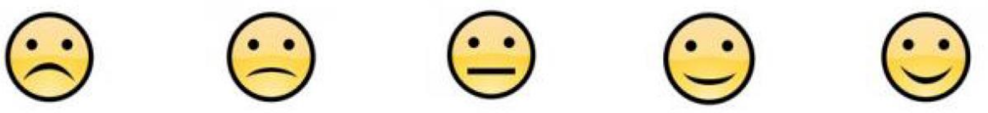

11. What do you like about the minigames?

12. What do you not like about the minigames?

13. How much did you like the worksheets you made?

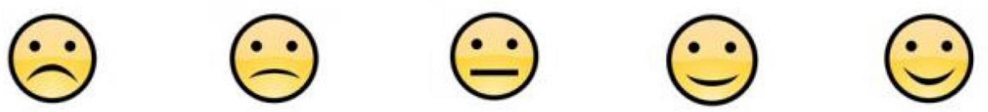

14. What did you like about the worksheets?

15. What did you not like about the worksheets?

16. What do you think about how many levels you had to play and how long it took you to play the levels?

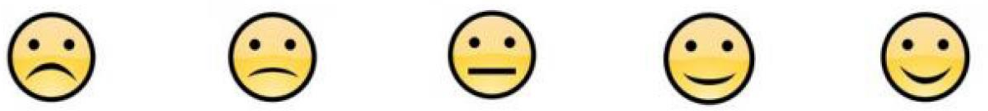

17. What do you think about playing the game Broodles alone, without your parents?

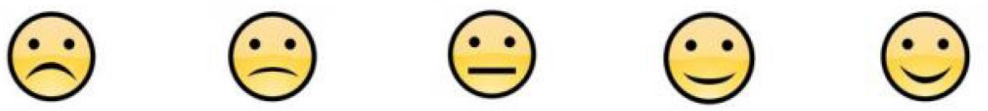

18. Was there anything in the game that you did not understand or thought was too difficult?

19. Was there anything in the game that you think was strange?

20. What did you learn from the game Broodles? And which part of the game or worksheets helped you with that?

    a) In general?

    b) About your thoughts and feelings?

    c) About understanding your brother or sister?

d) About how to deal with difficult situations?

21. What did you miss in the game or worksheets?

22. Would you recommend the game Broodles to other siblings? Why (not)?

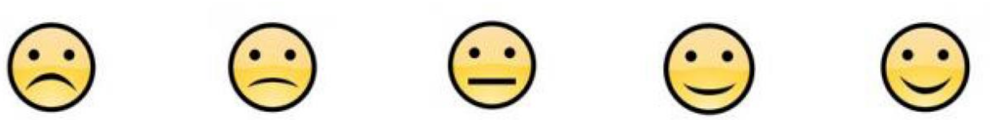

23. Is there anything else you want to say about the game Broodles?
