# Supplementary material for: Cultural applicability and desirability of ‘Broodles’: The first serious game intervention for siblings of children with disabilities
Source: PEC Innov. 2024 Mar 26;4:100277. doi: 10.1016/j.pecinn.2024.100277 (PMC11000116; doi:10.1016/j.pecinn.2024.100277)
Supplement: Supplementary Appendix A.4 — Final Evaluation Questionnaire 'Broodles' - Parent Version. [file mmc4.pdf]

Serious Game 'Broodles'

Final Evaluation Questionnaire – Parent Version

Hello! Thank you for participating in this study with your child. I will ask you a few questions about the serious game and parent guide. Could you answer them as thoroughly as possible? There are no right or wrong answers, it is about wat you think.

Evaluation questions about the game and work sheets

1. How many levels of the game did your child play?

2. What do you think about the game in general? Was it useful for you and your child that your child played this game?

3. What do you think about your child playing the game without your assistance or the assistance of a professional/mentor?

4. What did your child share with you about the game?

5. How many worksheets did your child make?

6. How many worksheets did your child make together with you?

7. What do you think about the worksheets in general? Was it useful for you and your child to complete the worksheets?

8. What do you think about the lengths of the work sheets? Was it feasible for you to make the work-sheets with your child in between the levels?

9. To what extend where the work sheets understandable to you and your child?

10. Was there anything in the game or worksheets that you thought was strange or odd?

11. Which parts of the game and worksheets did you think were most useful?

- ☐ Animations of the Broodles
- ☐ Quizzes about the animations, thoughts and feelings
- ☐ Emotion memory
- ☐ Videos of other siblings
- ☐ Helpful and non-helpful thoughts game
- ☐ Hidden object game
- ☐ Summary and tips on worksheets
- ☐ Tasks on the worksheets

Could you please explain why you think those are useful?

12. Which parts of the game and worksheets did you think were <u>not</u> useful?

- ☐ Animations of the Broodles
- ☐ Quizzes about the animations, thoughts and feelings
- ☐ Emotion memory
- ☐ Videos of other siblings
- ☐ Helpful and non-helpful thoughts game
- ☐ Hidden object game
- ☐ Summary and tips on worksheets
- ☐ Tasks on the worksheets

Could you please explain why you think those are <u>not</u> useful?

13. What do you think your child learned from the game and worksheets?

a) …in general?

b) …considering coping with thoughts and emotions?

c) …considering coping with challenging situations?

d) …considering understanding their brother or sister with a disability?

e) …considering feeling seen and heard?

f) …considering talking with me about his/her feelings and thoughts and challenging situations?

14. What did you miss in the game and worksheets?

About the parent guide

15. How much of the parent guide were you able to read?

- ❍ None
- ❍ Less than half
- ❍ Around half
- ❍ More than half
- ❍ All of it

16. What do you think about the parent guide in general? Was it useful for you?

17. What do you think about the length of the parent guide?

18. What do you think about the language use in the parent guide and to what extend was the parent guide understandable to you?

19. Was there anything in the parent guide that you thought was strange or odd?

20. Which information or tips in the parent guide were useful to you and why?

21. Which information or tips did you miss?

22. Did the parent guide provide sufficient information to support your child in playing the game?

23. Would you recommend this intervention to other families? Why (not)?

24. What do you think about the format of this intervention, being an individually played, standalone game?

25. What support do you think siblings and parents would need in addition to the game?
